# BlocTrain: Block-Wise Conditional Training and Inference for Efficient Spike-Based Deep Learning

**DOI:** 10.3389/fnins.2021.603433

**Published:** 2021-10-29

**Authors:** Gopalakrishnan Srinivasan, Kaushik Roy

**Affiliations:** Department of Electrical and Computer Engineering, Purdue University, West Lafayette, IN, United States

**Keywords:** deep SNNs, spike-based backpropagation, complexity-aware local training, greedy block-wise training, fast inference

## Abstract

Spiking neural networks (SNNs), with their inherent capability to learn sparse spike-based input representations over time, offer a promising solution for enabling the next generation of intelligent autonomous systems. Nevertheless, end-to-end training of deep SNNs is both compute- and memory-intensive because of the need to backpropagate error gradients through time. We propose BlocTrain, which is a scalable and complexity-aware incremental algorithm for memory-efficient training of deep SNNs. We divide a deep SNN into blocks, where each block consists of few convolutional layers followed by a classifier. We train the blocks sequentially using local errors from the classifier. Once a given block is trained, our algorithm dynamically figures out easy vs. hard classes using the class-wise accuracy, and trains the deeper block only on the hard class inputs. In addition, we also incorporate a hard class detector (HCD) per block that is used during inference to exit early for the easy class inputs and activate the deeper blocks only for the hard class inputs. We trained ResNet-9 SNN divided into three blocks, using BlocTrain, on CIFAR-10 and obtained 86.4% accuracy, which is achieved with up to 2.95× lower memory requirement during the course of training, and 1.89× compute efficiency per inference (due to early exit strategy) with 1.45× memory overhead (primarily due to classifier weights) compared to end-to-end network. We also trained ResNet-11, divided into four blocks, on CIFAR-100 and obtained 58.21% accuracy, which is one of the first reported accuracy for SNN trained entirely with spike-based backpropagation on CIFAR-100.

## 1. Introduction

Deep neural networks have achieved remarkable success and redefined the state-of-the-art performance for a variety of artificial intelligence tasks including image recognition (He et al., 2016), action recognition in videos (Simonyan and Zisserman, 2014a), and natural language processing (Bahdanau et al., 2014; Sutskever et al., 2014), among other tasks. We refer to modern deep neural networks as analog neural networks (ANNs) since they use artificial neurons (sigmoid, ReLU, etc.) that produce real-valued activations. ANNs attain superhuman performance by expending significant computational effort, which is believed to be much higher compared to the human brain. The quest for improved computational efficiency has led to the emergence of a new class of networks known as spiking neural networks (SNNs) (Maass, 1997), which are motivated by the sparse spike-based computation and communication capability of the human brain. The salient aspect of SNN is its ability to learn sparse spike-based input representations over time, which can be used to obtain higher computational efficiency during inference in specialized event-driven neuromorphic hardware (Merolla et al., 2014; Davies et al., 2018; Blouw et al., 2019).

Supervised training of SNNs is challenging and has attracted significant research interest in recent years (Lee et al., 2016, 2020; Bellec et al., 2018; Jin et al., 2018; Shrestha and Orchard, 2018; Wu et al., 2018; Neftci et al., 2019; Thiele et al., 2020). Error backpropagation algorithms, which are the workhorse for training deep ANNs with millions of parameters, suffer from scalability limitations when adapted for SNNs. It is well known that end-to-end training of feed-forward ANNs, using backpropagation, requires the activations of all the layers to be stored in memory for computing the weight updates. SNNs, by virtue of receiving input patterns converted to spike trains over certain number of time-steps, require multiple forward passes per input. As a result, spike-based backpropagation algorithms need to integrate error gradients through time (Neftci et al., 2019). The ensuing weight update computation requires the spiking neuronal activation and state (also known as membrane potential) to be stored across time-steps for the entire network. SNNs are typically trained for hundreds of time-steps to obtain high enough accuracy for visual image recognition tasks (Lee et al., 2020). Hence, end-to-end training of SNN using backpropagation through time (BPTT) requires much higher memory footprint over that incurred for training similarly sized ANN on Graphics Processing Units (GPUs) (Gruslys et al., 2016).

**Figure 1:**
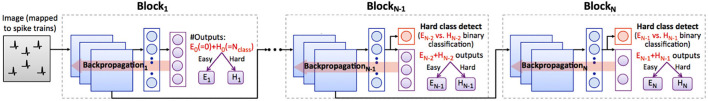
Illustration of BlocTrain methodology for block-wise input complexity aware training of deep SNNs. The blocks are trained sequentially using local losses from the respective classifiers. Each block *B*_*i*_ has a hard class detector that is trained to perform binary classification between the easy (*E*_*i*−1_) and the hard class instances (*H*_*i*−1_), as determined from the preceding block. The next block *B*_*i*+1_ is trained only on the hard class instances (*H*_*i*−1_). This process is repeated for every block, leading to fast learning with increasing block depth.

In this work, we propose input complexity driven block-wise training algorithm, referred to as *BlocTrain*, for incrementally training deep SNNs with reduced memory requirements compared to that incurred for end-to-end training. We divide a deep SNN into blocks, where each block consists of few convolutional layers followed by a local auxiliary classifier, as depicted in Figure 1. We train the blocks sequentially using local losses from the respective auxiliary classifiers. For training a particular block, we freeze the weights of the previously trained blocks and update only the current block weights using local losses from the auxiliary classifier. The proposed algorithm precludes the need for end-to-end backpropagation, thereby considerably reducing the memory requirements during training, albeit with overhead incurred due to the addition of a classifier per block. Next, we present a systematic methodology to determine the optimal SNN depth for a given application based on the target accuracy requirements. New blocks are added only if the accuracy of prior blocks (obtained on the validation set) is lower than the desired accuracy. Further, the newly appended blocks are trained only on the “hard” classes as summarized below. Once a particular block is trained, we subdivide the classes into “easy” and “hard” groups based on the class-wise accuracy on the validation set. We incorporate and train a HCD in the following block to perform binary classification between the “easy” and the “hard” class inputs. The next deeper block is now trained only on the hard class instances, as illustrated in Figure 1. Previous works on class complexity aware training built hierarchical classifier models, where the initial layers classify the inputs into coarse super-categories while the deeper layers predict the finer classes, which require end-to-end training and inference (Srivastava and Salakhutdinov, 2013; Yan et al., 2015; Panda et al., 2017a). On the other hand, *BlocTrain* significantly minimizes the training effort with increasing block depth due to gradual reduction in the number of output classes. During inference, we obtain improved computational efficiency by using the HCD per block to terminate early for easy class inputs and conditionally activate deeper blocks only for the hard class inputs. The higher inference efficiency is achieved with increased memory requirement owing to the use of nonlinear auxiliary classifiers. We demonstrate the capability of *BlocTrain* to provide improved accuracy as well as higher training (compute and memory) and inference (compute) efficiency relative to end-to-end approaches for deep SNNs on the CIFAR-10 and the CIFAR-100 datasets. Note that *BlocTrain*, although demonstrated in this work for SNNs, can be directly applied for ANNs to achieve efficient conditional training and inference. Overall, the key contributions of our work are as follows:

1. We propose a scalable training algorithm for deep SNNs, where the block-wise training strategy can help alleviate the larger memory requirement, which is bound by hardware limitations, and gradient propagation issues incurred by end-to-end training.
2. We present a systematic methodology to determine the optimal network size (in terms of number of layers) for a given dataset based on the accuracy requirements, since new layers are added and trained sequentially until the desired accuracy is achieved.
3. We improve the latency and compute efficiency during inference, which is achieved by using the HCD to exit early for the easy class instances and activate the deeper blocks only for the hard class instances.

## 2. Related Work

### 2.1. Local Training of Deep Neural Nets

Several approaches have been proposed to complement or address the challenge of end-to-end training of deep networks. Before the deep learning revolution (circa 2012), unsupervised layer-wise pre-training based on local loss functions was used to effectively initialize the weights of deep ANNs (stacked denoising autoencoder, deep belief nets, etc.) (Ivakhnenko and Lapa, 1965; Hinton and Salakhutdinov, 2006; Hinton et al., 2006; Bengio et al., 2007; Vincent et al., 2008; Erhan et al., 2010; Belilovsky et al., 2019). SNNs, on the contrary, have been pre-trained using spiking autoencoders (Panda and Roy, 2016) as well as more biologically plausible spike timing dependent plasticity (STDP) based localized learning rules (Masquelier and Thorpe, 2007; Diehl and Cook, 2015; Ferré et al., 2018; Kheradpisheh et al., 2018; Mozafari et al., 2018; Srinivasan et al., 2018; Tavanaei et al., 2018; Thiele et al., 2018; Lee et al., 2019; Srinivasan and Roy, 2019). Greedy layer-wise unsupervised training of SNNs has until now been demonstrated only for shallow networks (≤ 5 layers), yielding considerably lower than state-of-the-art accuracy on complex datasets, for instance, ~71% on CIFAR-10 (Panda and Roy, 2016; Ferré et al., 2018). Some works have also proposed supervised pre-training of deep networks using losses generated by auxiliary classifier per layer (Marquez et al., 2018). However, pre-training is followed by end-to-end backpropagation to attain improved accuracy and generalization for both ANNs (Erhan et al., 2010; Dong et al., 2018) and SNNs (Lee et al., 2018).

Very few works use only the local losses generated by the layer-wise auxiliary classifier to train deep nets (Kaiser et al., 2018; Mostafa et al., 2018; Nøkland and Eidnes, 2019). Mostafa et al. (2018) found that layer-wise training using only the local discriminative loss caused the accuracy of a 10-layer deep ANN to saturate after the sixth layer with an accuracy of ~83%, which is lower than that (~87%) achieved with end-to-end error backpropagation on CIFAR-10. Nøkland and Eidnes (2019) supplemented the local discriminative loss using similarity matching loss to converge to the accuracy provided by end-to-end backpropagation. Alternatively, Jaderberg et al. (2017) proposed incorporating a decoupled neural network at every layer (or every few layers) of the original deep ANN to produce synthetic gradients that are trained to match the true gradients obtained with global backpropagation.

### 2.2. Fast Inference for Deep Nets

Fast inference methods use auxiliary classifiers at various intermediate layers of a deep network and terminate inference sequentially at different classifiers based on the input complexity (Panda et al., 2016, 2017b; Teerapittayanon et al., 2016; Huang et al., 2018). The end-to-end network and classifiers can be either trained independent of each other (Panda et al., 2016, 2017b) or co-optimized to minimize the weighted cumulative loss of all classifiers (Teerapittayanon et al., 2016; Huang et al., 2018). Inference is terminated at the earlier classifiers for easy inputs and the deeper classifiers for hard inputs, resulting in improved latency and computational efficiency.

*BlocTrain* differs from prior works in the following respects:

1. We introduce auxiliary classifiers at the granularity of blocks of convolutional layers and train the blocks sequentially using only the local discriminative loss.
2. We train the deeper blocks only on hard classes, which are automatically deduced by *BlocTrain* based on the class-wise accuracy of the earlier blocks on the validation set.
3. *BlocTrain* leads to fast inference by detecting instances belonging to easy or hard classes learnt during training. Prior approaches classify the instances as easy or hard irrespective of their class labels. Our inference method incurs lower training effort with increasing block depth while the latter approach requires all the blocks to be trained on the entire dataset.

## 3. Spike-Based Input Representation, Neurons, and BPTT

**Figure 2:**
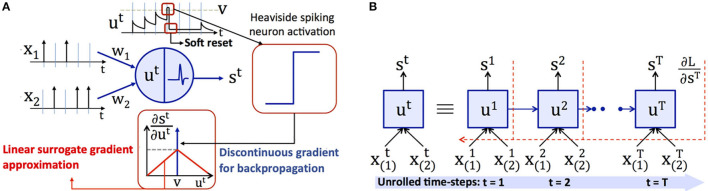
**(A)** Spike-based input representation, and membrane potential dynamics, soft reset behavior, and piece-wise linear surrogate gradient for a leaky integrate and fire (LIF) neuron. An LIF neuron integrates the weighted sum of input spikes ($X_{\left(i\right)}^{t}$) with the synaptic weights (*w*_*i*_) and emits a spike (*s*^*t*^) when its membrane potential (*u*^*t*^) exceeds the firing threshold (*v*). In the event of a spike, the membrane potential is reduced (or soft reset) by an amount equal to the firing threshold. During backpropagation, the discontinuous Dirac delta gradient ($\frac{\partial s^{t}}{\partial u^{t}}$) is replaced by a continuous surrogate gradient approximation. **(B)** Illustration of backpropagation through time for a spiking neuron, where in the output loss ($\frac{\partial L}{\partial s^{t}}$) is accumulated over *T* simulation time-steps.

The unique attributes of deep SNNs over ANNs are spike-based input coding and neuronal nonlinearity, which facilitate temporal information processing. For vision tasks, the input pixels are converted to Poisson-distributed spike trains firing at a rate proportional to the corresponding pixel intensities, as described in Heeger (2000) and shown in Figure 2A. The number of time-steps (latency) determine the training as well as inference efficiency, and is in the order of few hundreds of time-steps (Jin et al., 2018; Lee et al., 2020). At any given time, the weighted sum of the input spikes gets integrated into the membrane potential of “soft reset” leaky integrate and fire (LIF) neuron (Diehl et al., 2016), whose dynamics are described by


(1) $$\begin{array}{r} \begin{array}{c} u^{t+1}=\alpha u^{t}+\sum_{i}w_{i}x_{i}^{t}-vs^{t}\\ s^{t}=\Theta \left(\frac{u^{t}}{v}-1\right) \end{array} \end{array}$$

where *u* is the membrane potential, superscript *t* indicates the time-step, α is the rate of leak of membrane potential, *w*_*i*_ and *x*_*i*_ are the weight and spike train of *i*th input neuron, *v* is the firing threshold, *s* is the spike output, and Θ is the Heaviside step function. The LIF neuron produces a spike when its membrane potential exceeds the firing threshold. At the instant of a spike, the membrane potential is “soft reset” by reducing its value by an amount equal to the threshold voltage, as described in Equation (1). The “soft reset” mechanism carries over the residual potential above threshold at the firing instants to the following time-step, thereby minimizing the information loss during forward propagation.

Backpropagation is performed by unrolling the network and integrating the losses over time as depicted in Figure 2B. The weight update (Δ*w*_*i*_) is computed as described by


(2) $$\begin{array}{r} \Delta w_{i}=\underset{t}{\sum }\frac{\partial L}{\partial w_{i}^{t}}=\underset{t}{\sum }\frac{\partial L}{\partial s^{t}}\frac{\partial s^{t}}{\partial u^{t}}\frac{\partial u^{t}}{\partial w_{i}^{t}} \end{array}$$

where *L* is the loss function [Mean Squared Error (MSE) loss, cross-entropy loss, etc.] that measures the deviation of the actual network output from the target (class label for image recognition tasks). The partial derivative of the LIF neuron output with respect to the membrane potential, $\frac{\partial s^{t}}{\partial u^{t}}$, is the derivative of the Heaviside function specified in Equation (1). The LIF output derivative is described by the Dirac delta function, $\delta \left(\frac{u^{t}}{v}-1\right)$, which is not defined at the spiking instants (*t* ∈ ℕ^+^|*u*^*t*^ = *v*) and is zero elsewhere. The Dirac delta derivative is not suitable for backpropagation since it precludes the effective backward flow of error gradients. The discontinuous derivative is replaced by a smooth function, known as surrogate gradient, around the spiking instants (Bellec et al., 2018; Shrestha and Orchard, 2018; Zenke and Ganguli, 2018; Roy et al., 2019). We use the piece-wise linear surrogate gradient (Bellec et al., 2018), which is specified as


(3) $$\begin{array}{r} \frac{\partial s^{t}}{\partial u^{t}}\approx \gamma \text{ Max}\left(0,1-|\frac{u^{t}}{v}-1|\right) \end{array}$$

where γ (< 1) is the gradient dampening factor. The linear surrogate gradient is maximum at the spiking instants and linearly decreases elsewhere based on the absolute difference between the membrane potential and threshold as depicted in Figure 2A. We refer the readers to Neftci et al. (2019) for a survey of surrogate gradient approximations proposed in literature.

## 4. BlocTrain Training and Inference Algorithm

### 4.1. Block-Wise Complexity-Aware Training

In this section, we describe the block-wise complexity-aware incremental algorithm for memory-efficient training of deep SNNs. We divide a deep spiking network into blocks, where each block is composed of few convolutional and/ or pooling layers followed by a classifier, as illustrated in Figure 1. We use nonlinear classifiers, consisting of an additional hidden layer before the final softmax layer. Hence, the location of the classifiers needs to be chosen judiciously for achieving improved training efficiency with minimal parameters overhead. Algorithm 1 details the presented block-wise training methodology. We train the first block *B*_1_ on the entire training set using surrogate gradient-based BPTT (Algorithm 2), which is discussed later in this section. We then compute its class-wise accuracy on the validation set. If the accuracy of a class is lower (higher) than a pre-determined “hardness threshold,” the class is grouped as a hard (easy) class. The following block *B*_2_ is trained on the easy and hard class instances of *B*_1_ (entire training set) with frozen *B*_1_ weights. The softmax units of *B*_2_ are trained with cross-entropy loss computed using the class labels. In addition, we introduce an HCD, which is a binary neuron with sigmoidal activation function. The HCD unit is trained with sigmoid cross-entropy loss to perform binary classification between the easy and the hard class inputs. We then determine the class-wise accuracy of the combined (*B*_1_ + *B*_2_) network using fast inference method (refer to Algorithm 3), detailed in section 4.2. Based on the class-wise accuracy of *B*_2_, we further divide the hard classes of *B*_1_ into finer easy and hard classes. The next block *B*_3_ is then trained on the finer easy and hard class instances of *B*_2_, which are basically the hard class instances of *B*_1_. In general, a given block *B*_*i*_ is trained on the easy and hard inputs of *B*_*i*−1_ (same as the hard inputs of *B*_*i*−2_) with fixed *B*_1_ … *B*_*i*−1_ weights, as described in Algorithm 1. BlocTrain leads to higher compute and memory efficiency compared to end-to-end methods. In addition, we also show (in section 5) that residual connections between the blocks enable the deeper blocks to learn better representations, leading to higher accuracy.

**Algorithm 1:**
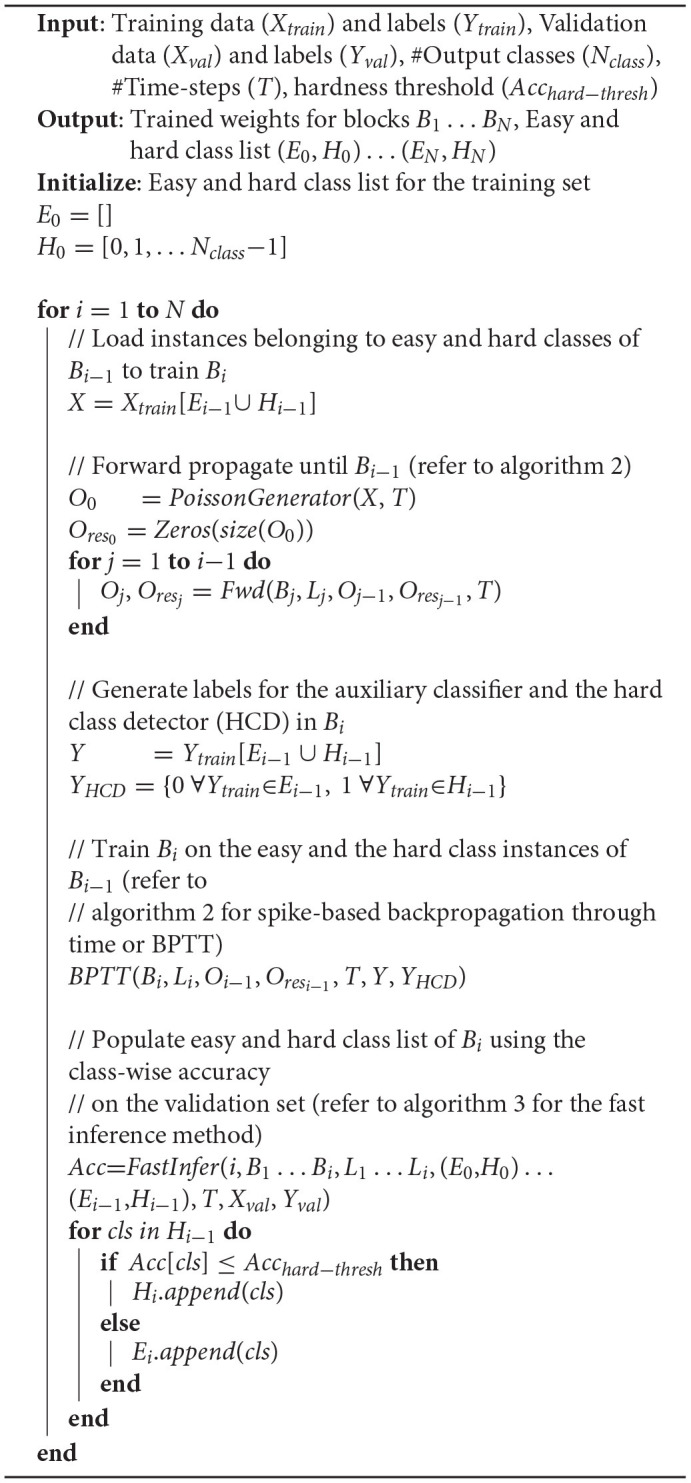
Block-wise training for SNN with *N* blocks *B*_1_ … *B*_*N*_, where block *B*_*i*_ has *L*_*i*_ layers.

Next, we detail the surrogate gradient-based BPTT algorithm used for training the SNN blocks. The convolutional and linear layers of the SNN are followed by LIF nonlinearity, as described in Algorithm 2. During forward pass, Heaviside step function is applied to the LIF neuron membrane potentials for generating spike inputs to the following layer at every time instant. In addition, the membrane potentials and spiking activations are stored for computing and backpropagating the surrogate gradients during the BPTT phase. The average pooling layers, on the contrary, are followed by integrate-and-fire nonlinearity (α = 1 in Equation 1). This is because the pooled neurons do not encode complex temporal dynamics, and spike based on the average firing rate of the LIF neurons located past the preceding convolutional layer. During the BPTT phase, the output gradients are passed through the pooling layers. The output layer, consisting of the softmax and the HCD units, is not subjected to spike-based nonlinearity to enable precise computation of the output loss directly using the membrane potential of the output neurons. The final loss, which is the sum total of the cross-entropy loss of the softmax units and sigmoid cross-entropy loss of the binary HCD unit, is minimized using BPTT.

**Algorithm 2:**
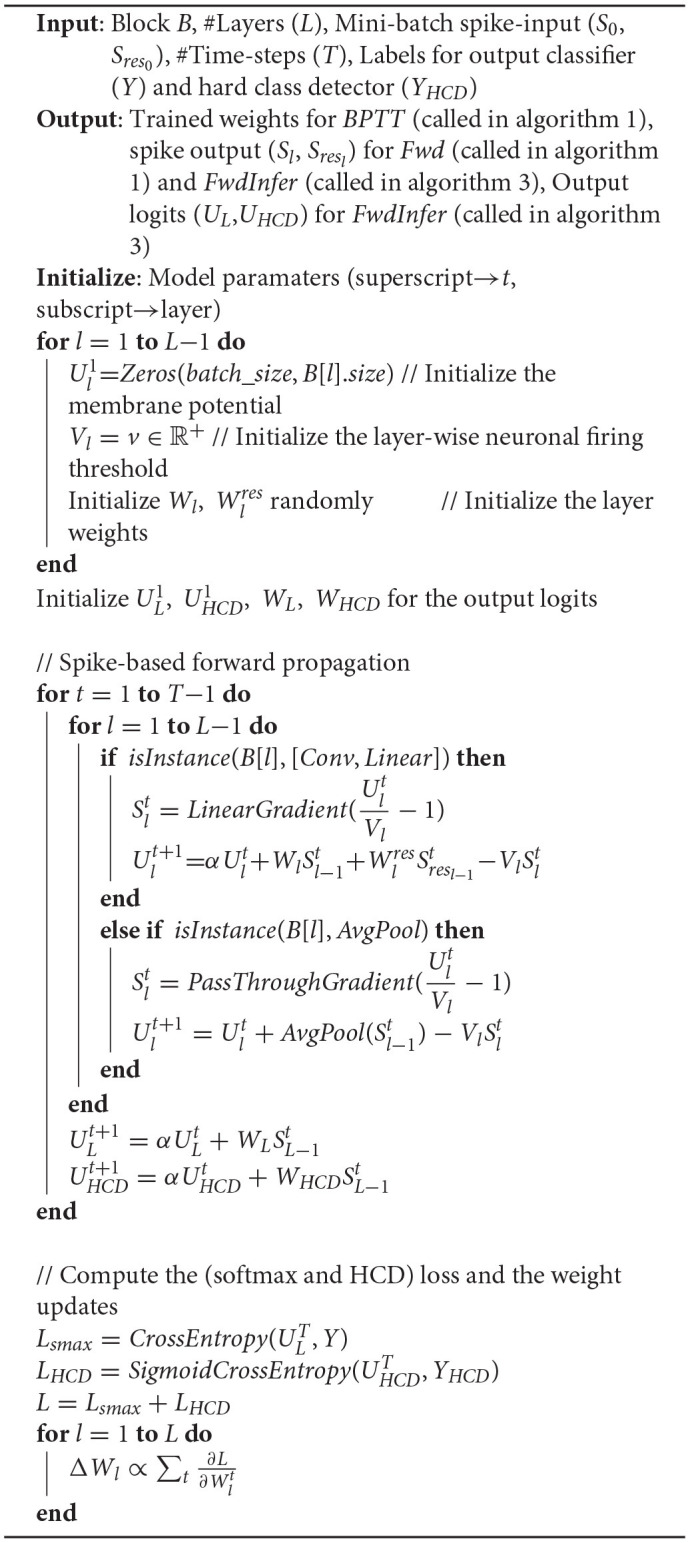
Mini-batch (with *batch*_*size*) spike-based backpropagation through time (BPTT).

### 4.2. Fast Inference With Early Exit

BlocTrain, on account of introducing intermediate classifiers (or exit branches), leads to fast inference, with early exit, for deep SNNs as described in Algorithm 3. The inference is terminated at a given block *B*_*i*_ using the softmax and hard class prediction probabilities as the confidence measure for the classifier and the HCD, respectively. Note that the softmax probabilities at *B*_*i*_ are obtained using the cumulative sum of the corresponding logits with their counterparts in the previous block *B*_*i*−1_. We find that combining the classifier outputs by summing up the respective logits improves the final prediction accuracy since the blocks are trained independently. Our method of combining the individual classifier outputs to boost the final accuracy is similar to adaptive boosting (Freund and Schapire, 1995), which combines multiple weak classifiers into a strong one. Inference is terminated at *B*_*i*_ under the following conditions.

1. if the classifier exhibits high confidence, that is, if the classifier prediction probability is higher than a pre-determined confidence threshold (θ_*conf*_);
2. if the HCD is low in confidence, that is, if the HCD prediction probability is lower than hard-class confidence threshold (θ_*high*_), in which case it is not favorable to activate the subsequent block.

**Algorithm 3:**
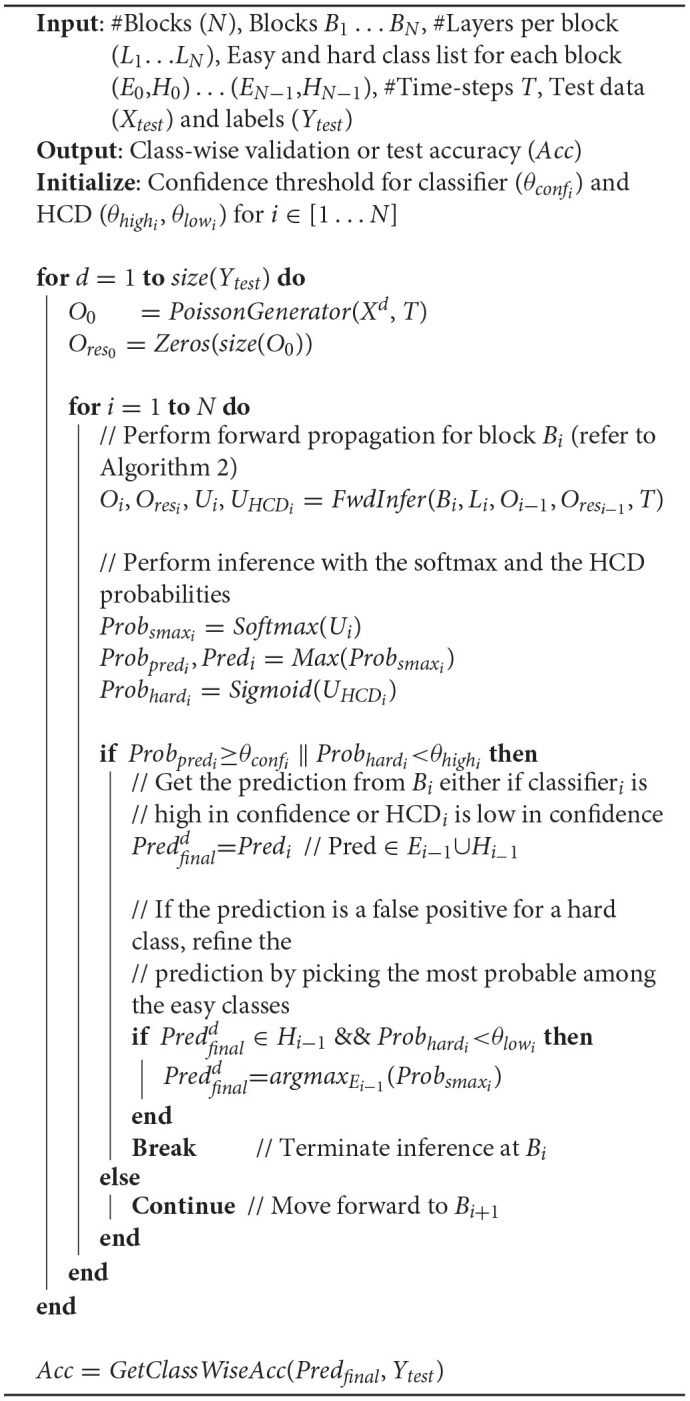
Fast inference, with early exit, algorithm for spiking neural networks (SNNs).

Additionally, if the prediction at *B*_*i*_ belongs to the hard class list of *B*_*i*−1_ while the HCD probability is much smaller than easy-class detection threshold (θ_*low*_), the original prediction is possibly a false positive for the predicted hard class. In this case, the original prediction at *B*_*i*_ is refined by selecting the one with maximum probability among the softmax units, which belong exclusively to the easy class list of *B*_*i*−1_. Only in the event that the classifier is low in confidence and the HCD is high in confidence, the next deeper block *B*_*i*+1_ is activated. This process is repeated for the all the blocks sequentially beginning from the first block, leading to improved computational efficiency during inference, with memory overhead incurred due to the use of nonlinear intermediate classifiers and for storing the binary spiking activations to be fed to the following block. Higher the number of instances classified at the early exit branches, larger is the computational efficiency benefit with reduced memory overhead compared to end-to-end inference.

## 5. Results

### 5.1. Experimental Setup

**Figure 3:**
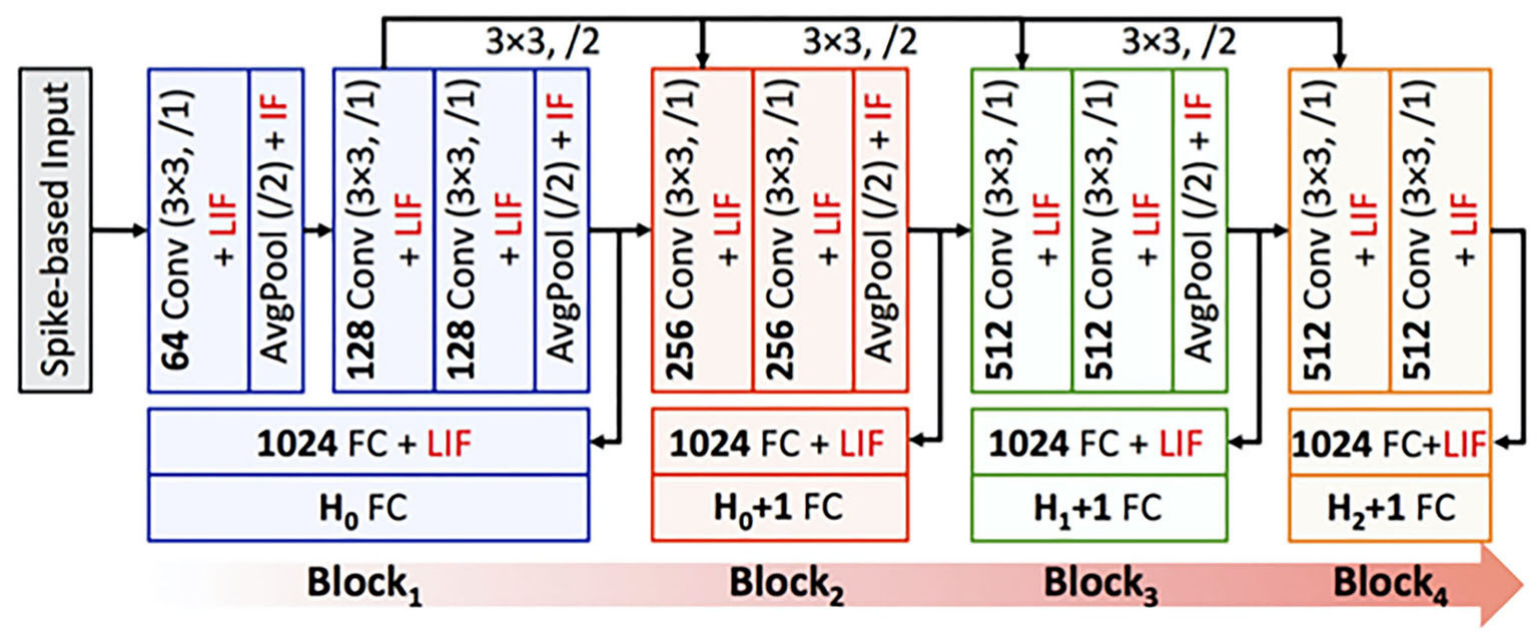
ResNet-11 spiking neural network (SNN), similar to the end-to-end topology presented in Lee et al. (2020), used to validate BlocTrain. The first 3 blocks make up ResNet-9 SNN. Block_1_ is trained on all the classes (*H*_0_). Any other block_*i*_ is trained on the hard classes of block_*i*−2_ (*H*_*i*−2_), and has an additional hard class detector (HCD) binary unit. The number of output feature maps, kernel size, stride, and spiking nonlinearity are specified for all the layers in each block of the ResNet SNN analyzed in this work.

We demonstrate the efficacy of BlocTrain for ResNet-9 (on CIFAR-10), and ResNet-11 and VGG-16 (on CIFAR-100), which are among the deepest models trained entirely using spike-based BPTT algorithms (Lee et al., 2020). ResNet-9 (ResNet-11) is divided into 3 (4) blocks as illustrated in Figure 3. The input image pixels are normalized to zero mean and unit variance, and mapped to Poisson spike trains firing at a maximum rate of 1,000 *Hz* over 100 time-steps. We generate positive or negative spikes, based on the sign of the normalized pixel intensities, firing at a rate proportional to the absolute value of the intensities as described in Sengupta et al. (2019). For most experiments in this work unless mentioned otherwise, the original CIFAR-10 or CIFAR-100 training set, consisting of 50,000 images, is split into a training subset of 40,000 images and validation subset of 10,000 images. Training is performed on the training subset (for 125 epochs) using Adam optimizer (Kingma and Ba, 2014), with mini-batch size of 64, and learning rate of 2e-4 for the first two blocks and 1e-4 for the rest of the blocks as well as the baseline end-to-end model. Once a given block is trained, the class-wise accuracy on the validation subset is used to determine the easy and the hard classes. The baseline model is obtained by removing the local classifiers shown in Figure 3. The accuracy of the trained models is reported on the test set of 10,000 images. The code for SNN training and inference, using BlocTrain and end-to-end method, is uploaded as Supplementary Material.

### 5.2. ResNet-9 SNN on CIFAR-10

**Figure 4:**
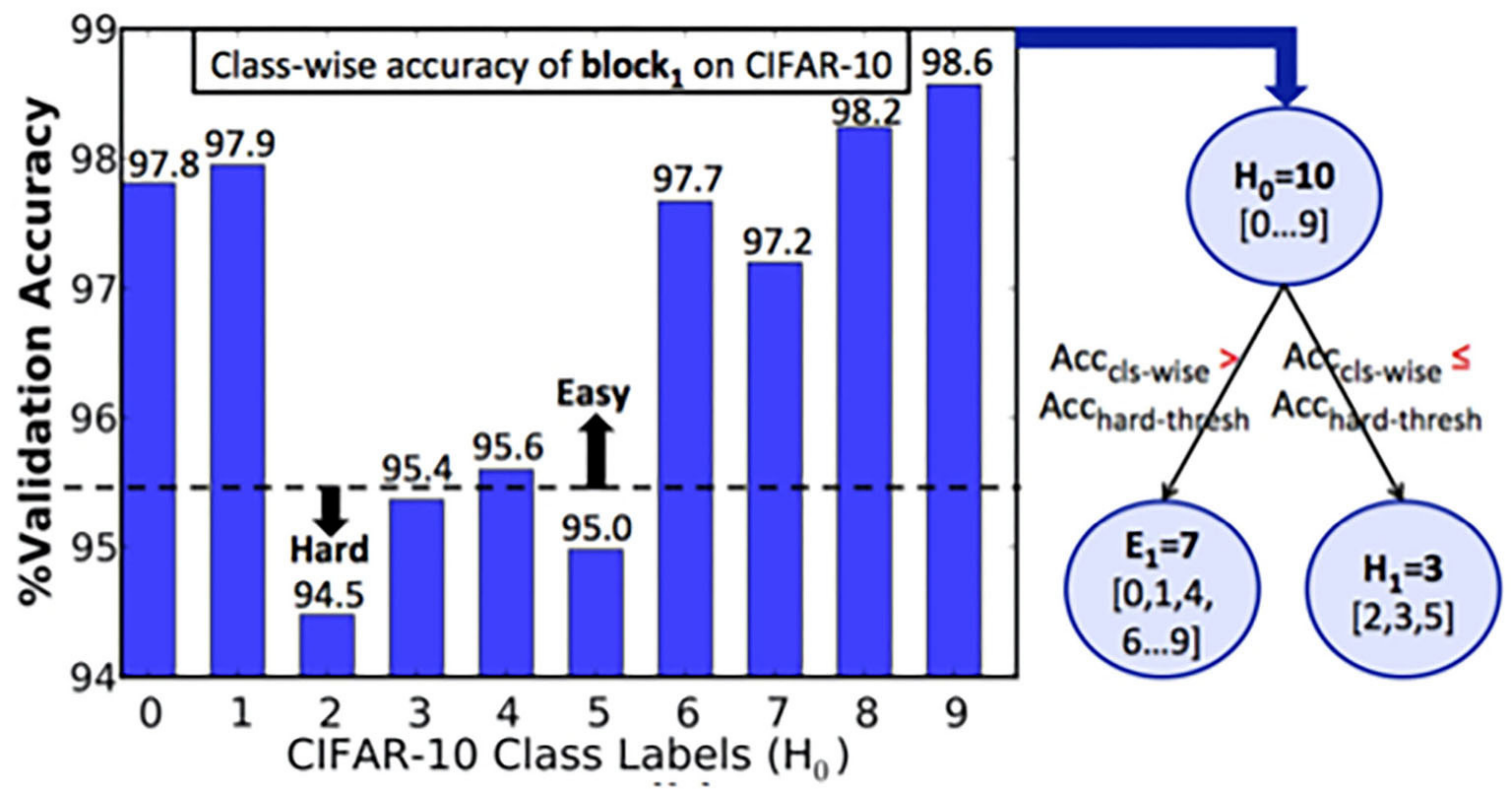
Algorithm to determine easy vs. hard classes based on the class-wise accuracy of ResNet9-block_1_ on the CIFAR-10 validation subset. If the class-wise accuracy, *Acc*_*cls*−*wise*_, is lesser (greater) than the hardness threshold, *Acc*_*hard*−*thresh*_, the class is categorized as a hard (easy) class.

We trained the first block *B*_1_ of ResNet-9 SNN on the CIFAR-10 training subset. The class-wise accuracy provided by *B*_1_ (on the validation set) at the end of training is shown in Figure 4. Based on the hard-class accuracy threshold (*Acc*_*hard*−*thresh*_) of 95.5%, BlocTrain automatically categorized the original CIFAR-10 classes into 7 easy (*E*_1_) and 3 hard classes (*H*_1_), as depicted in Figure 4. We then trained the classifier of the next block *B*_2_ on all the 10 classes, and the binary HCD unit for distinguishing between the easy (*E*_1_) and the hard groups (*H*_1_). Following the training of *B*_2_, the last block *B*_3_ was trained on only the 3 hard classes of *B*_1_. We first present the training efficiency benefits offered by BlocTrain and then discuss the inference accuracy-efficiency trade-off.

The training efficiency of BlocTrain over end-to-end approach is quantified using the memory requirement for performing BPTT. For training a block *B*_*i*_, BlocTrain requires only the spiking activations and membrane potentials of *B*_*i*_ to be stored across time-steps in addition to the weights of all the blocks until *B*_*i*_. Note that the classifier of previous blocks are not necessary for training the current block, and hence, they are ignored for estimating the memory requirement for the current block. Also, the spiking activations, being binary, consumes 32× smaller memory footprint than that for the weights and membrane potentials. End-to-end method, on the other hand, requires the weights, potentials, and activations of the entire network for performing BPTT. Our analysis shows that BlocTrain incurs 1.32× -2.95× lower memory requirement relative to end-to-end BPTT. In addition, we also find that the BlocTrain memory requirement decreases until *B*_2_ after which it slightly increases, albeit much lower than end-to-end BPTT. The higher memory requirement for *B*_3_ stems from an increase in the block parameters as shown in Figure 3. Finally, our experiments indicate that the training time reduces with block depth beginning from *B*_3_. *B*_2_, on account of being fed by *B*_1_ and trained on all the classes, incurs slightly longer training time relative to *B*_1_. Overall, ResNet-9 SNN trained using BlocTrain on a Nvidia GeForce GTX GPU with 11178MiB memory capacity incurs 1.13× slowdown in training time per epoch over end-to-end training when the same mini-batch size is used for both methods. Section 6.4.2 details the training time incurred by BlocTrain, relative to end-to-end training, on different training hardware configurations. Aside from memory efficiency, BlocTrain offers the following benefits, as quantified and discussed in the subsequent paragraphs.

1. BlocTrain leads to stable training convergence by effectively circumventing the gradient propagation issues plaguing end-to-end SNN training approaches, leading to higher accuracy.
2. BlocTrain, by virtue of estimating the optimal SNN size based on dataset complexity and using early exit inference strategy, offers improved latency and computational efficiency during inference.

**Figure 5:**
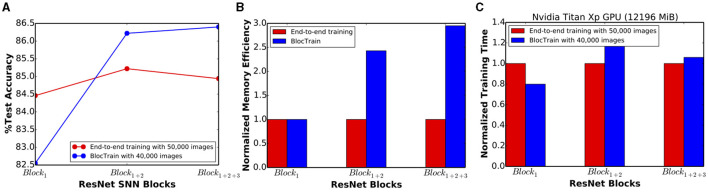
**(A)** Test accuracy, **(B)** normalized training memory efficiency, and **(C)** normalized training time offered by BlocTrain over end-to-end training for ResNet-5 (*Block*_1_), ResNet-7 (*Block*_1+2_), and ResNet-9 (*Block*_1+2+3_) spiking neural networks (SNNs).

ResNet-9 SNN (trained using BlocTrain) offered 86.4% test accuracy when inference was performed, as described in Algorithm 3, using the classifier confidence threshold (θ_*conf*_) set to unity. Next, in order to quantify the impact of inter-block residual connections, we trained a VGG9-like network (ResNet-9 in Figure 3 without residual connections) using BlocTrain. The VGG9-like SNN provided lower accuracy of 85.5%, which indicates that residual connections between the blocks enable the deeper blocks to learn better high-level representations. The test accuracy of 86.4% provided by ResNet-9 is roughly 1.5% higher than that achieved with end-to-end network training (without the intermediate classifiers). This is a counterintuitive, albeit interesting, finding since end-to-end training of deep ANNs has been shown to outperform local training using intermediate classifiers (Marquez et al., 2018; Mostafa et al., 2018). For deep SNNs, stable convergence of end-to-end training, by eliminating the vanishing gradient phenomenon, largely depends on proper layer-wise threshold initialization and choosing the “right” surrogate gradient parameters. BlocTrain, by using divide-and-conquer based incremental training method, effectively circumvents the initialization dilemma by limiting the gradient flow to few layers at any given time. In order to evaluate the training convergence properties of BlocTrain with increasing block depth relative to end-to-end training, we trained 3 different networks, namely ResNet-5 (*Block*_1_), ResNet-7 (*Block*_1+2_), and ResNet-9 (*Block*_1+2+3_). Note that we used the same parameters for the thresholds and the surrogate gradients, as suggested by Bellec et al. (2018) and Lee et al. (2020), respectively, for BlocTrain as well as end-to-end training. Figure 5A indicates that end-to-end training yields higher accuracy than BlocTrain for *Block*_1_, which can be attributed to the fact that BlocTrain uses a smaller training subset (refer to section 5.1), while end-to-end training uses the entire training set. However, as more blocks are appended, BlocTrain offers superior accuracy than end-to-end training despite using a smaller training subset. In fact, end-to-end training causes slight accuracy degradation for *Block*_1+2+3_ compared to *Block*_1+2_ network, as depicted in Figure 5A. The improved accuracy offered by BlocTrain is achieved with higher memory efficiency, as illustrated in Figure 5B. Training time, on the contrary, increases with block depth for BlocTrain over end-to-end training when equivalent mini-batch size is used for both approaches, as shown in Figure 5C. The increase in training time is primarily caused by the need to perform multiple forward passes for the earlier blocks to train deeper blocks. We refer the readers to section 6.4.2 for comparative analysis of training time under different mini-batch size considerations.

**Figure 6:**
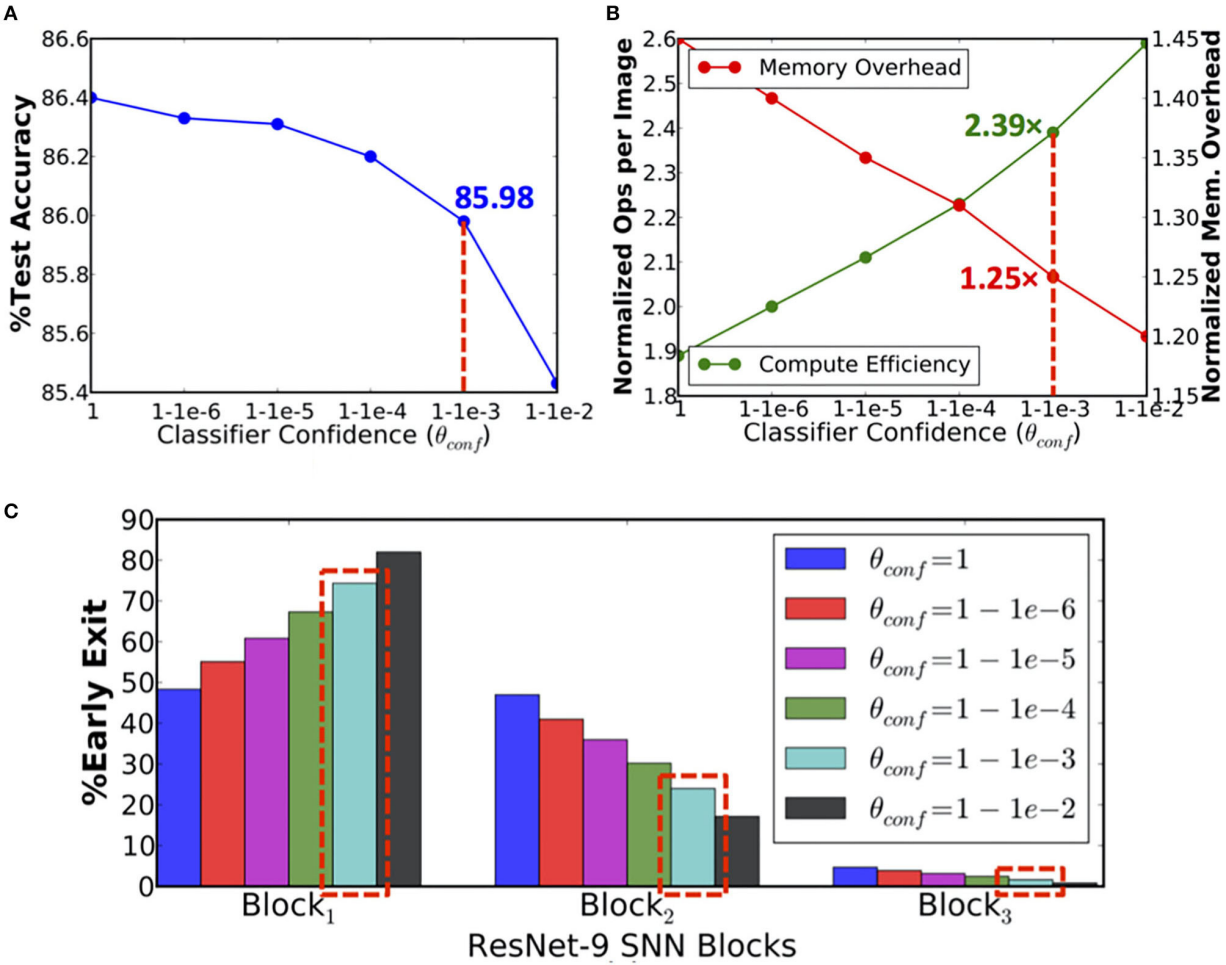
**(A)** Test accuracy, **(B)** computational efficiency in terms of the normalized number of synaptic operations and memory overhead per inference, and **(C)** percentage of exiting inputs per block vs. classifier confidence thresholds (θ_*conf*_) for ResNet-9 spiking neural network (SNN) on CIFAR-10.

During inference, ResNet-9 offers 1.89× higher compute efficiency over the baseline model due to early exit strategy. The compute efficiency is estimated based on the number of operations (in the convolutional and linear layers) per inference, averaged over the test set. However, ResNet-9 also incurs 1.45× memory overhead to store and access the nonlinear fully connected classifier parameters and block-wise spiking activations per inference. Figure 6 indicates that as the classifier confidence thresholds are relaxed to enable more instances to exit at *B*_1_, the overall compute efficiency increases with commensurate reduction in the memory overhead. We obtain 2.39× higher compute efficiency with 1.25× memory overhead per inference relative to the baseline network with <0.5% drop in accuracy, as shown in Figures 6A,B.

### 5.3. ResNet-11 SNN on CIFAR-100

**Figure 7:**
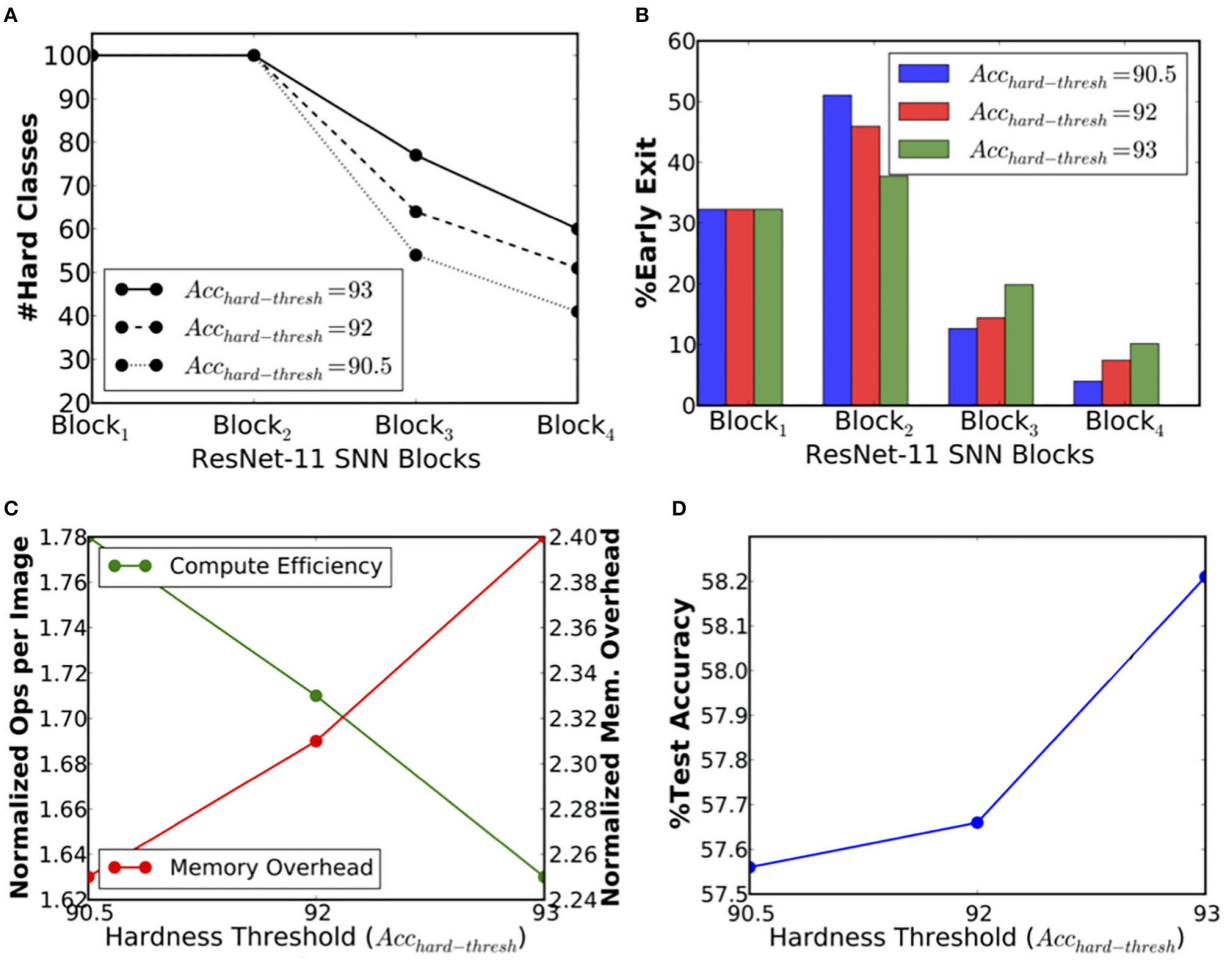
**(A)** Number of output classes per block, **(B)** percentage of exiting inputs per block, **(C)** compute efficiency in terms of the normalized number of synaptic operations and memory overhead per inference, and **(D)** test accuracy vs. hardness threshold (*Acc*_*hard*−*thresh*_) for ResNet-11 spiking neural network (SNN) trained on the CIFAR-100 dataset.

In the previous section 5.2, we demonstrated that BlocTrain could dynamically figure out the easy and the hard classes during the course of training. However, in CIFAR-10, there was clear separation between the easy and the hard classes. Hence, we could not analyze what impact would different choices for hard classes have on the training and the inference efficiency. We set forth to answer this question for ResNet-11 on CIFAR-100. Once *B*_1_ (*B*_2_) was trained, we generated three different sets of hard classes for *B*_3_ (*B*_4_) by setting the hardness threshold (*Acc*_*hard*−*thresh*_ in Algorithm 1) to 90.5, 92, and 93%, respectively. Higher the hardness threshold, larger is the number of hard classes for the deeper layers, and vice versa, as shown in Figure 7A. For instance, hardness threshold of 90.5% is relatively easy to satisfy in the earlier blocks, resulting in fewer hard classes for the deeper layers. On the other hand, a higher hardness threshold of 93% leads to much more hard classes for the deeper layers. The training effort for the deeper layers directly corresponds to the chosen hardness threshold. Higher the hardness threshold, longer is the training time for the deeper layers.

During inference (θ_*conf*_ set to 0.9999), we found that the number of instances classified at *B*_1_ was the same for all the three ResNet-11 models, which is expected since the HCD is only pertinent beyond *B*_1_. Beginning from *B*_2_, the models with higher hardness threshold of 92% and 93% were pushing more inputs to the deeper layers, *B*_3_ and *B*_4_, while the one with lowest threshold was classifying a larger fraction of the inputs at *B*_2_, as shown in Figure 7B. As a result, ResNet-11 with hardness threshold of 90.5% has the highest compute efficiency during inference (1.78×) followed by the others, as depicted in Figure 7C. Also, it has the lowest test accuracy (57.56%) relative to that (58.21%) offered by ResNet-11 with the highest threshold, as shown in Figure 7D. However, the accuracy increase is only 0.65%, which indicates that the deeper layers could not significantly improve the accuracy for the hard classes. This could be an artifact of the CIFAR-100 dataset, which has only 500 instances per class. Nevertheless, our analysis indicates that the test accuracy of 58.21%, offered by BlocTrain for ResNet-11 SNN on CIFAR-100, is ~6% higher relative to that obtained with end-to-end training. The superior accuracy offered by BlocTrain is a testament to its ability to scale to deeper SNNs for complex datasets. Finally, we note that ResNet-11 incurs >2× parameters overhead, as shown in Figure 7C, due to the inclusion of four nonlinear classifiers. The overhead can be reduced by merging the *B*_1_ and *B*_2_ classifiers since >70% of the instances are classified at *B*_2_, and by using linear classifiers.

### 5.4. VGG-16 SNN on CIFAR-100

**Figure 8:**
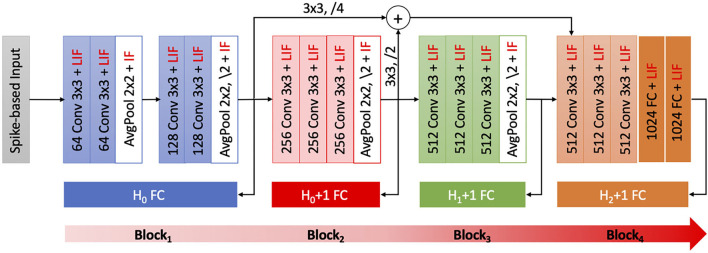
Illustration of VGG-16 spiking neural network (SNN) divided into 4 blocks, where each block is trained sequentially using BlocTrain. The first block is trained on all the *H*_0_ classes. Any subsequent block, *Block*_*i*_, is trained on the hard classes of *Block*_*i*−2_ (denoted by *H*_*i*−2_ in the figure). The final block receives residual connections from *Block*_1_ and *Block*_2_ to improve the training efficiency. The number of output feature maps and kernel size are specified for all the blocks. The stride is set to unity unless explicitly mentioned otherwise.

**Figure 9:**
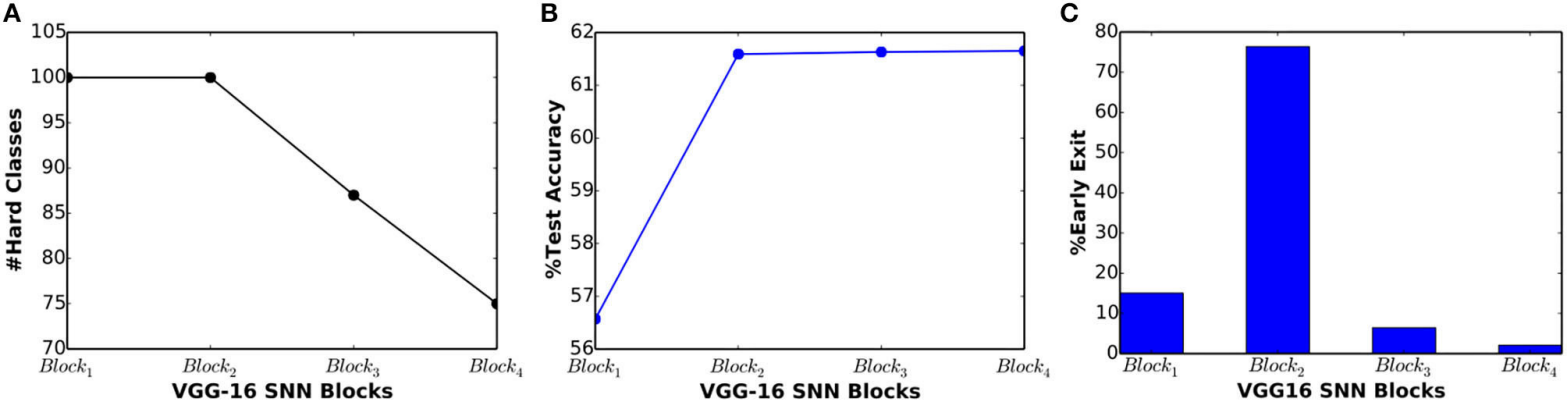
**(A)** Number of output classes, **(B)** test accuracy, and **(C)** percentage of exiting inputs vs. block depth for VGG-16 spiking neural network (SNN), trained on the CIFAR-100 dataset.

In order to demonstrate the scalability of BlocTrain to deeper SNNs, we trained VGG-16 architecture (Simonyan and Zisserman, 2014b) divided into 4 blocks, as illustrated in Figure 8. Each block is equipped with a simple linear classifier without any hidden layers so as to reduce the parameter overhead imposed by BlocTrain. In addition, the final block (*Block*_4_) receives residual inputs from *Block*_1_ and *Block*_2_ for addressing the issue of vanishing spikes to deeper blocks of a network. Also, the firing threshold of the convolutional layers in *Block*_4_ needed to be tuned for ensuring efficient spike propagation and gradient backpropagation. The firing threshold of the remaining blocks is set to unity. Thus, BlocTrain offers a prior to suitably initialize the firing threshold of deeper blocks. On the contrary, threshold initialization remains a challenge for end-to-end training methods. Too high a firing threshold leads to vanishing spikes, thereby, necessitating longer simulation time-steps to achieve competitive accuracy. Too low a threshold causes exploding spikes, which could negatively impact training convergence and accuracy. All the blocks are trained on the entire CIFAR-100 training set. The test set is used to deduce the easy and the hard classes post the training of each block. The first two blocks are trained on all the CIFAR-100 classes, while *Block*_3_ and *Block*_4_ are trained on 87 and 75 hard classes, respectively, as shown in Figure 9A. The test accuracy, depicted in Figure 9B, increases until *Block*_2_ and nearly saturates for deeper blocks. VGG-16 SNN achieves best test accuracy of 61.65%, where in majority of inferences are terminated in the earlier blocks, as shown in Figure 9C. We already demonstrated the ability of BlocTrain to provide higher accuracy than end-to-end training, in sections 5.2 and 5.3, when the same input coding, spiking nonlinearity, and backpropagation algorithm (and the associated hyperparameters) are used for both methods. Future works could improve the accuracy of deeper blocks in large networks by introducing additional diversity during the training of deeper blocks. For large datasets, this can be achieved by partitioning the dataset across the earlier and deeper blocks. In addition, neural architecture search (Elsken et al., 2019) could be used to determine the optimal number of hard classes for deeper layers.

## 6. Discussion

### 6.1. BlocTrain Hyperparameters Heuristics

In this section, we present the heuristics for setting the BlocTrain hyperparameters, namely, the hard-class accuracy threshold, also referred to as the class hardness threshold (*Acc*_*hard*−*thresh*_ in Algorithm 1) and the softmax classifier confidence threshold (θ_*conf*_ in Algorithm 3). The choice of these hyperparameters directly impacts the trade-off among memory overhead, compute efficiency, and test accuracy, as illustrated in Figures 6, 7. Our experiments using ResNet-9 on CIFAR-10 (Figure 6) and ResNet-11 on CIFAR-100 (Figure 7) establishes the following key heuristics and trends on the hardness threshold. First, the hardness threshold is experimentally found to be bounded within the range [μ_*acc*_−σ_*acc*_, μ_*acc*_+σ_*acc*_], where μ_*acc*_ is the mean and σ_*acc*_ is the standard deviation of the class-wise accuracies on the validation set to obtain favorable trade-off among memory overhead, compute efficiency, and test accuracy. Second, higher the hardness threshold, larger is the memory overhead, lower is the compute efficiency, and better is the test accuracy. For ResNet-9 on CIFAR-10, we fixed the hardness threshold to 95.5%, which is roughly equal to the experimental lower bound of μ_*acc*_−σ_*acc*_, where μ_*acc*_ and σ_*acc*_ are 96.79 and 1.43%, respectively, calculated using the class-wise accuracies reported in Figure 4. For CIFAR-10, using the lower bound on the hardness threshold provided favorable memory overhead-test accuracy trade-off since there were only 10 classes with clear separation between the easy and the hard classes, as illustrated in Figure 4.

On the other hand, for ResNet-11 on CIFAR-100, we experimented with hardness thresholds of 90.5–93%, which is roughly in the range of μ_*acc*_ to μ_*acc*_+σ_*acc*_. Setting the hardness threshold closer to μ_*acc*_ categorized roughly 50 classes as hard (refer to Figure 7A) based on the validation accuracy of the first trained block in ResNet-11. Lowering the hardness threshold any further would provide <50% of the total number of classes for the deeper block. Hence, we did not investigate hardness thresholds much lower than μ_*acc*_. On the contrary, setting the hardness threshold to 93% (~μ_*acc*_+σ_*acc*_) categorized close to 80 classes as hard, leading to higher memory overhead and lower compute efficiency relative to that achieved with hardness threshold of 92% (~μ_*acc*_+0.5*σ_*acc*_). Hence, for any network to be trained on a complex dataset such as CIFAR-100 with a mix of easy and hard classes, setting the hardness threshold closer to μ_*acc*_+0.5*σ_*acc*_ should yield favorable trade-offs among memory overhead, compute efficiency, and accuracy. However, if all the class probabilities are similar and the class-wise validation accuracies are high, it implies that the dataset has mostly “easy” classes, and hence, the hardness threshold can be set to the lower bound. On the other hand, if the class probabilities are similar and the class-wise validation accuracies are low, then the dataset has predominantly “hard” classes, and hence, the hardness threshold could be set closer to the upper bound. Thus, the hardness threshold, *per se*, does not introduce additional complexity during the training process. As far as the softmax classifier confidence threshold (θ_*conf*_) is concerned, we investigated values ranging from ln(10^−2^) to ln(10^−6^) in logarithmic scale. Our experimental results across the CIFAR-10 and the CIFAR-100 datasets indicate that θ_*conf*_ of ln(10^−3^) or ln(10^−4^) yields favorable compute efficiency-accuracy trade-off. Hence, the choice of θ_*conf*_ should not require extensive experimentation to identify the optimal threshold.

### 6.2. Blocking Strategy for Deeper SNNs

For the SNNs analyzed in this work, namely, ResNet-9 and ResNet-11, we divided the network at the granularity of a residual block and, consequently, inserted an auxiliary classifier for every residual block. Much deeper networks such as VGG-19 (Simonyan and Zisserman, 2014b) and ResNet-34 (He et al., 2016) could be divided at the granularity of few VGG and residual blocks, respectively, to minimize the overhead stemming from the extra softmax layer while limiting the gradient flow to a few layers for stable training using spike-based BPTT. A more principled approach could be to take into account the memory and computational cost of adding a classifier after a certain block and the fraction of instances reaching the block (obtained from the HCD of the prior classifier block) for guiding the placement process as proposed in Panda et al. (2017b). Such a principled methodology will help avoid inserting too many classifiers, and at the same time help determine the optimal network size for a given dataset based on the accuracy requirements.

### 6.3. Comparison With Early Inference

The proposed BlocTrain method categorizes the classes as hard or easy, and trains deeper blocks only on the hard class instances. Inference is terminated at the earlier blocks for easy class instances while the deeper blocks are activated only when hard class instances are detected. It is important to note that BlocTrain attributes uniform hardness (or significance) to all instances of any given class. In practice, the hardness might not be uniform across all instances of a class, as noted in prior works (Panda et al., 2016; Teerapittayanon et al., 2016), which categorized individual instance as hard or easy irrespective of the general difficulty of the corresponding class. Therefore, we set forth to compare the efficacy of BlocTrain with respect to baseline method, designated as BlocTrain-base, wherein every block is trained on all the classes. Inference is terminated at a particular block based on the classifier confidence, that is, if the classifier prediction probability is higher than a specified confidence threshold (θ_*conf*_). The BlocTrain-base method effectively classifies easy instances, belonging to any class, at the earlier blocks and activates the deeper blocks only for hard instances. For the proposed BlocTrain method, the original CIFAR-10 or CIFAR-100 dataset, containing 50,000 images, is split into training set of 40,000 images and validation set of 10,000 images. The validation set is used to subdivide the classes into easy and hard groups, as noted in section 5.1. On the contrary, the entire dataset is used for BlocTrain-base since each of the blocks is trained on all the classes. The classifier confidence threshold is set to unity for all the blocks, which causes inference to be terminated at a given block only if the prediction is obtained with 100% confidence. Setting the confidence threshold to unity yields the best test accuracy since it encourages more instances to be classified at the deeper blocks.

**Figure 10:**
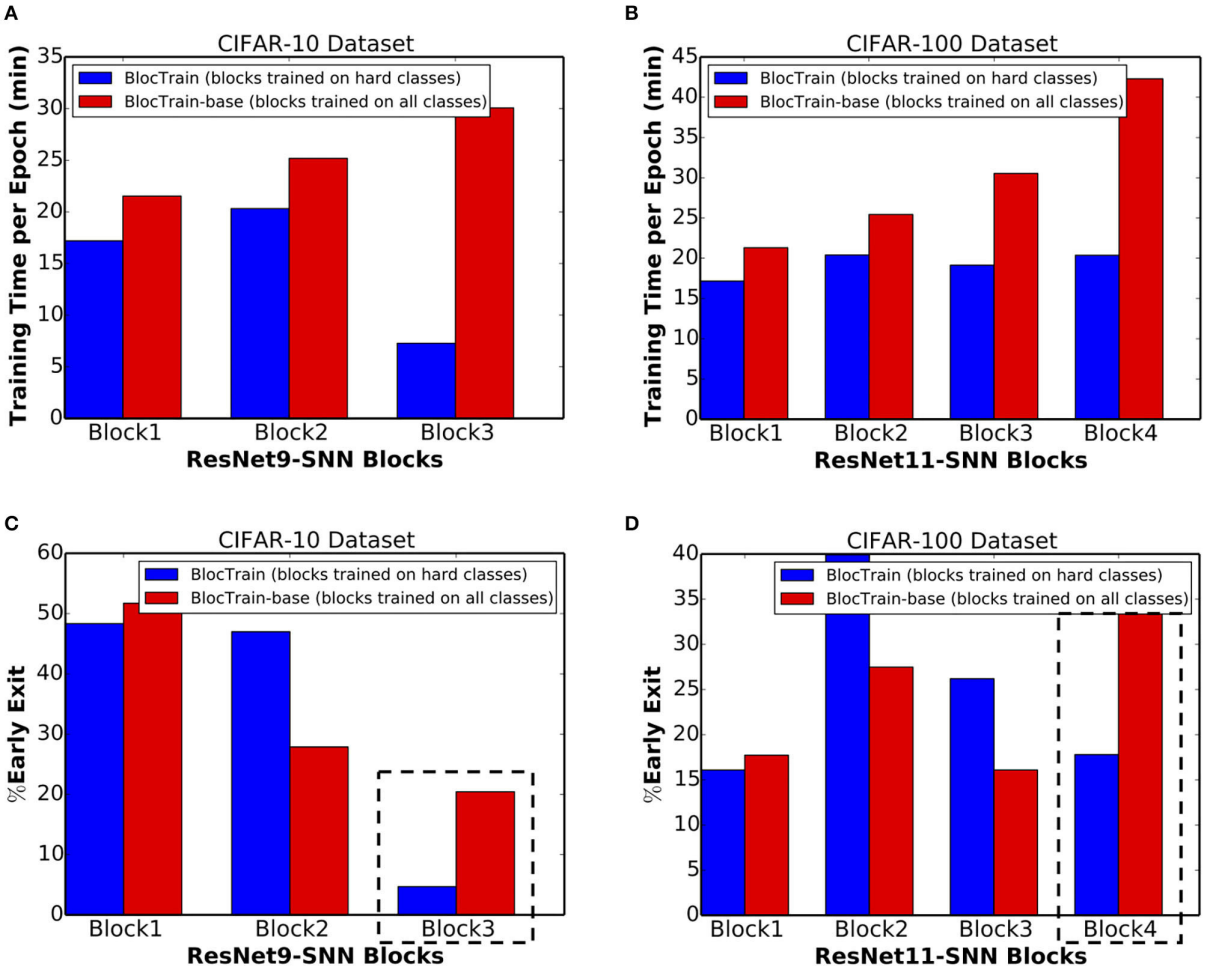
**(A)** Training time per epoch incurred by successive blocks of ResNet-9 spiking neural network (SNN), trained on CIFAR-10 using BlocTrain, wherein deeper blocks are trained on hard classes, and BlocTrain-base, wherein deeper blocks are trained on all classes. **(B)** Training time per epoch incurred by successive blocks of ResNet-11 SNN, trained using BlocTrain and BlocTrain-base methods, on the CIFAR-100 dataset. **(C)** Percentage of exiting inputs per block for ResNet-9 SNN, trained using BlocTrain and BlocTrain-base methods, on the CIFAR-10 dataset. **(D)** Percentage of exiting inputs per block for ResNet-11 SNN, trained using BlocTrain and BlocTrain-base methods, on the CIFAR-100 dataset.

We first present the training efficiency results followed by inference accuracy-efficiency trade-off provided by BlocTrain compared to the BlocTrain-base method. BlocTrain offers reduced or comparable training time (or effort) with increasing block depth. On the contrary, the training time increases steadily with block depth for BlocTrain-base, as shown in Figures 10A,B for ResNet-9 (on CIFAR-10) and ResNet-11 (on CIFAR-100), respectively. BlocTrain-base incurs higher training effort compared to BlocTrain due to the following couple of reasons. First, BlocTrain-base uses the entire training dataset while BlocTrain divides the original dataset into separate training and validation sets. Second, BlocTrain-base trains every block on all the class instances while BlocTrain uses only the hard class instances for deeper blocks. Despite the higher training effort, BlocTrain-base offers 88.31% test accuracy for ResNet-9 SNN on CIFAR-10, which is higher than an accuracy of 86.4% provided by BlocTrain. For ResNet-11 SNN on CIFAR-100, BlocTrain-base offers 62.03% accuracy, which is even higher compared to an accuracy of 58.33% provided by BlocTrain. The higher accuracy provided by BlocTrain-base can be attributed to the following factors. First, BlocTrain-base uses the entire original dataset for training all the blocks. Second, BlocTrain-base enables the harder instances in every class to be executed at the deeper blocks, resulting in higher accuracy. On the contrary, BlocTrain classifies both the easy and the hard instances of an “easy” class in the earlier blocks, leading to relatively inferior accuracy. The superior accuracy offered by BlocTrain-base is obtained with 8.5% and 7.8% higher computational effort (in terms of number of synaptic operations per inference) for ResNet-9 (on CIFAR-10) and ResNet-11 (on CIFAR-100), respectively. This is because BlocTrain-base classifies a larger fraction of hard instances at the ultimate block, as shown in Figures 10C,D. In summary, BlocTrain-base offers higher accuracy compared to BlocTrain, albeit, with longer training time and higher computational effort during inference.

### 6.4. Comparison With End-to-End Training

#### 6.4.1. Accuracy Comparison

Deep SNNs consisting of 7–11 layers, trained using end-to-end spike-based backpropagation approaches, have been shown to achieve >90% accuracy on CIFAR-10, as shown in Table 1. These networks are trained end-to-end with different surrogate gradient approximations, for the discontinuous spiking nonlinearity, than the one used in this work. The various surrogate gradient-based backpropagation approaches can be readily integrated into BlocTrain to further improve its efficacy. In the ANN domain, Mostafa et al. (2018) performed layer-wise training of 10-layer deep ANN using only the local discriminative loss and reported best accuracy of ~83% on CIFAR-10. BlocTrain, on account of block-wise rather than layer-wise training, provides much higher accuracy on CIFAR-10. On the other hand, very few works have reported CIFAR-100 accuracy for SNN trained entirely with spike-based BPTT, as noted in Table 2. Thiele et al. (2020) reported 64.69% accuracy for 8-layer deep SNN, wherein the training was performed on an equivalent ANN using the proposed SpikeGrad algorithm. Interestingly, Ledinauskas et al. (2020) trained ResNet-50 using end-to-end spike-based backpropagation and obtained 58.5% accuracy, which is comparable to that provided by ResNet-11 and lower than that obtained with VGG-16, trained using BlocTrain.

**Table 1:** Accuracy of spiking neural network (SNN) trained using BlocTrain and end-to-end spike-based backpropagation through time (BPTT) methods, and SNN/analog neural network (ANN) trained using only the local losses, on the CIFAR-10 dataset.

**Model**	**Training method**	**Dataset size**	**%Accuracy**
CIFARNet w/ 7 layers (Wu et al., 2019)	End-to-end STBP (Wu et al., 2018)	50,000	90.53
ResNet-9 (Lee et al., 2020)	End-to-end Spike BP	50,000	90.35
SNN w/ 8 layers (Thiele et al., 2020)	End-to-end ANN-based SpikeGrad	50,000	89.72
ResNet-11 (Ledinauskas et al., 2020)	End-to-end Spike BP	50,000	90.2
VGG-16 (Rathi et al., 2020)	ANN-SNN and end-to-end STDB	50,000	91.13
VGG-16 (Zhou et al., 2020)	Direct end-to-end BP	50,000	92.68
SNN w/ 4 layers (Panda and Roy, 2016)	Local AutoEncoder	50,000	70.16
ANN w/ 10 layers (Mostafa et al., 2018)	Local training	50,000	~83
**ResNet-9 (our work)**	**BlocTrain**	**40,000**	**86.4**
**ResNet-9 (our work)**	**BlocTrain-base**	**50,000**	**88.31**

*The bold values are used to highlight the results reported in this work over prior works*.

**Table 2:** Accuracy of spiking neural network (SNN) trained using BlocTrain and end-to-end spike-based backpropagation through time (BPTT) methods on the CIFAR-100 dataset.

**Model**	**Training method**	**Dataset size**	**%Accuracy**
SNN w/ 8 layers (Thiele et al., 2020)	End-to-end ANN-based SpikeGrad	50,000	64.69
VGG-11 (Rathi et al., 2020)	ANN-SNN and end-to-end STDB	50,000	67.87
ResNet-50 (Ledinauskas et al., 2020)	End-to-end Spike BP	50,000	58.5
**ResNet-11 (our work)**	**BlocTrain**	**40,000**	**58.21**
**ResNet-11 (our work)**	**BlocTrain-base**	**50,000**	**62.03**
**VGG-16 (our work)**	**BlocTrain**	**50,000**	**61.65**

*The bold values are used to highlight the results reported in this work over prior works*.

Finally, we note that prior works have demonstrated much deeper SNNs, with competitive accuracy, for CIFAR-10, CIFAR-100, and ImageNet datasets, using either standalone ANN–SNN conversion (Rueckauer et al., 2017; Sengupta et al., 2019; Han and Roy, 2020; Han et al., 2020) or a combination of ANN–SNN conversion and spike-based BPTT methods (Rathi et al., 2020; Wu et al., 2020). The hybrid approach initializes the weights and firing thresholds of the SNN using the trained weights of the corresponding ANN, and then performs incremental spike-based BPTT to fine-tune the SNN weights. Such a hybrid SNN training methodology can be incorporated into BlocTrain to achieve further improvements in accuracy on standard vision datasets. However, the primary objective of our work is to improve the training and inference capability of deep SNN for event-driven spatiotemporal inputs, such as those produced by dynamic vision sensors (Lichtsteiner et al., 2008), which could potentially require exclusive spike-based training to precisely learn the input temporal statistics. We demonstrated higher accuracy using BlocTrain over end-to-end spike-based BPTT methods on CIFAR-10 and CIFAR-100 data, mapped to spike trains, which indicates the capability of BlocTrain to scale to deep SNNs for complex event-based inputs.

#### 6.4.2. Training Time Comparison

**Figure 11:**
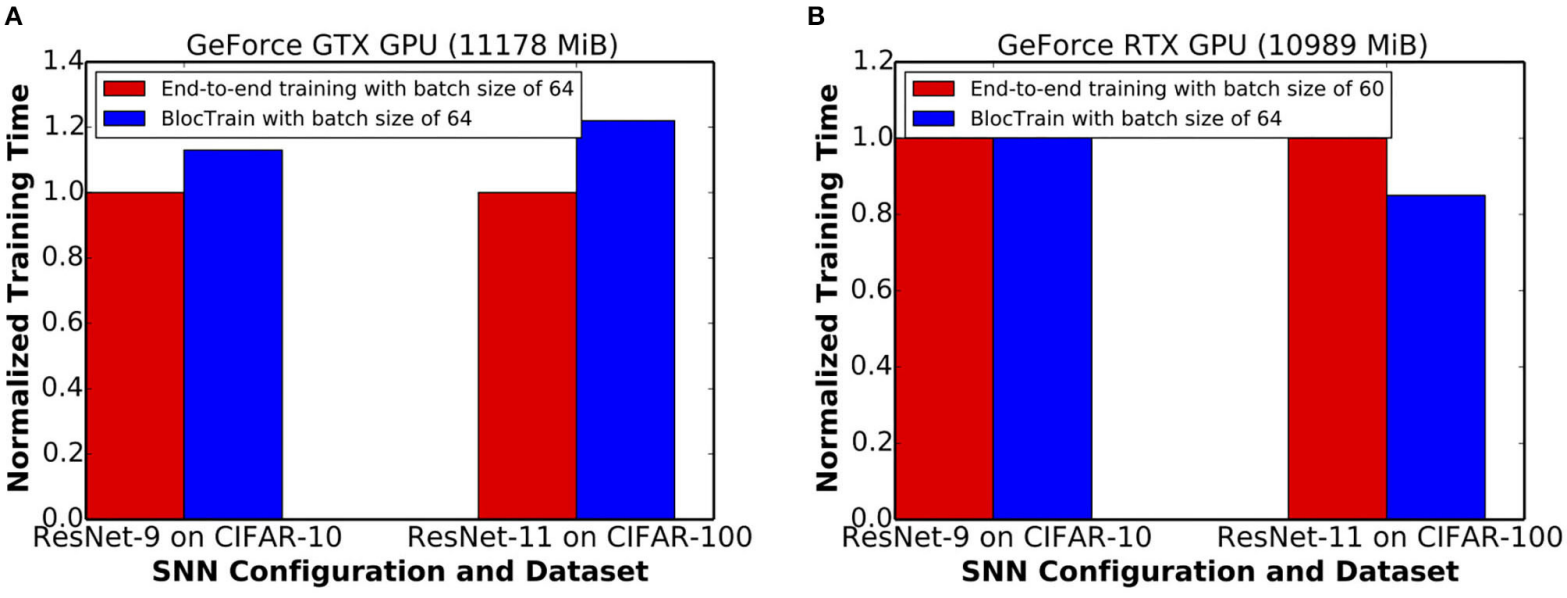
**(A)** Normalized training time per epoch of ResNet-9 and ResNet-11, trained using BlocTrain, relative to end-to-end training on the **(A)** GeForce GTX GPU and **(B)** GeForce RTX GPU.

The training time incurred by BlocTrain, relative to end-to-end training, depends on the training hardware memory limitations. We evaluated the training time on two different GPU configurations, namely, Nvidia GeForce GTX and RTX GPUs. The GeForce GTX GPU, on account of higher memory capacity, could sustain the same batch size of 64 for both BlocTrain and end-to-end training methods. Figure 11A indicates that ResNet-9 SNN and ResNet-11 SNN, trained using BlocTrain on the GeForce GTX GPU, incurs 1.13× and 1.22× longer training time, respectively, compared to end-to-end training. The longer training time incurred by BlocTrain over end-to-end training, when the same batch size is used for both the methods, can be attributed to the following twofold reasons.

1. BlocTrain requires multiple forward passes per block during training, as detailed below for ResNet-9 SNN, consisting of 3 blocks. *Block*_1_ incurs 3 separate forward passes for individually training each of the blocks. The second block incurs 2 forward passes to train *Block*_2_ and *Block*_3_. The third and final block entails a single forward pass to train *Block*_3_. On the other hand, end-to-end training incurs only a single forward pass for all the blocks.
2. Each block in the original network has an additional nonlinear classifier that needs to be trained.

Next, we evaluated the training times for BlocTrain and end-to-end training on the GeForce RTX GPU, which has relatively lower memory capacity. BlocTrain, by virtue of higher memory efficiency, could be used to train both ResNet-9 and ResNet-11 with a batch size of 64. End-to-end training, on account of hardware memory limitation, necessitated the batch size to be reduced to 60. Smaller batch size leads to higher number of batches (or iterations) per training epoch. As a result, BlocTrain incurs comparable training time for ResNet-9 and 0.85× shorter training time for ResNet-11 SNN over end-to-end training. For much deeper networks, the larger memory requirement needed for end-to-end training could either preclude SNN training or cause the batch size to be much smaller than that used for BlocTrain, depending on the hardware memory limitations. In the case that end-to-end training uses comparatively smaller batch size, BlocTrain would be both training time and memory efficient, as shown in Figure 11B.

## 7. Conclusion

End-to-end training of deep SNNs is memory-inefficient due to the need to perform error BPTT. In this work, we presented BlocTrain, which is a scalable block-wise training algorithm for deep SNNs with reduced memory requirements. During training, BlocTrain dynamically categorized the classes into easy and hard groups, and trained the deeper blocks only on the hard class inputs. In addition, we introduced a hard class detector per block to enable fast inference with early exit for the easy class inputs and conditional activation of deeper blocks only for the hard class inputs. Thus, BlocTrain provides a principled methodology to determine the optimal network size (in terms of number of layers) for a given task, depending on the accuracy requirements. We demonstrated BlocTrain for deep SNNs trained using spike-based BPTT, on the CIFAR-10 and the CIFAR-100 datasets, with higher accuracy than end-to-end training method. Future works could further improve the effectiveness of BlocTrain by using more complex methods for determining the hard classes, such as considering the false positives and negatives aside from the class-wise accuracy. Also, the local discriminative loss, which is used to separately train the individual blocks, could be augmented with other local losses as proposed in Nøkland and Eidnes (2019). Finally, well-established methods like neural architecture search could be used for selecting the BlocTrain hyperparameters such as the hardness threshold.

## Data Availability Statement

Publicly available datasets were analyzed in this study. This data can be found here: https://www.cs.toronto.edu/~kriz/cifar.html.

## Author Contributions

GS wrote the manuscript and performed the simulations. KR helped with writing of the manuscript, developing the concepts, and conceiving the experiments. Both authors contributed to the article and approved the submitted version.

## Funding

This work was supported in part by the Center for Brain Inspired Computing (C-BRIC), one of the six centers in JUMP, a Semiconductor Research Corporation (SRC) program sponsored by DARPA, by the Semiconductor Research Corporation, the National Science Foundation, and the DoD Vannevar Bush Fellowship.

## Conflict of Interest

The authors declare that the research was conducted in the absence of any commercial or financial relationships that could be construed as a potential conflict of interest.

## Publisher's Note

All claims expressed in this article are solely those of the authors and do not necessarily represent those of their affiliated organizations, or those of the publisher, the editors and the reviewers. Any product that may be evaluated in this article, or claim that may be made by its manufacturer, is not guaranteed or endorsed by the publisher.
